# Photo-Oxygenation as a New Therapeutic Strategy for Neurodegenerative Proteinopathies by Enhancing the Clearance of Amyloid Proteins

**DOI:** 10.3389/fnagi.2022.945017

**Published:** 2022-06-23

**Authors:** Ikumi Tomizawa, Hanako Nakagawa, Youhei Sohma, Motomu Kanai, Yukiko Hori, Taisuke Tomita

**Affiliations:** ^1^Laboratory of Neuropathology and Neuroscience, Graduate School of Pharmaceutical Sciences, The University of Tokyo, Tokyo, Japan; ^2^Department of Medicinal Chemistry, School of Pharmaceutical Sciences, Wakayama Medical University, Wakayama, Japan; ^3^Laboratory of Synthetic Organic Chemistry, Graduate School of Pharmaceutical Sciences, The University of Tokyo, Tokyo, Japan

**Keywords:** photo-oxygenation, Alzheimer disease, amyloid-beta, tau, microglia, amyloid, immunotherapy

## Abstract

Alzheimer disease (AD) is associated with the aggregation of two amyloid proteins: tau and amyloid-β (Aβ). The results of immunotherapies have shown that enhancing the clearance and suppressing the aggregation of these two proteins are effective therapeutic strategies for AD. We have developed photocatalysts that attach oxygen atoms to Aβ and tau aggregates *via* light irradiation. Photo-oxygenation of these amyloid aggregates reduced their neurotoxicity by suppressing their aggregation both *in vitro* and *in vivo*. Furthermore, photo-oxygenation enhanced the clearance of Aβ in the brain and microglial cells. Here, we describe the effects of photo-oxygenation on tau and Aβ aggregation, and the potential of photo-oxygenation as a therapeutic strategy for AD, acting *via* microglial clearance.

## Introduction

**Figure 1:**
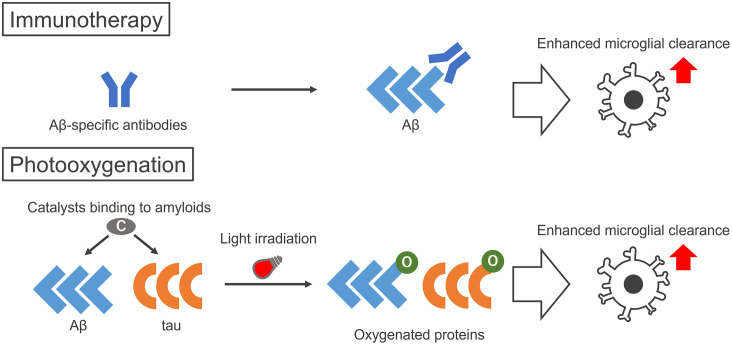
Illustration of the therapeutic strategies to enhance microglial clearance of amyloid proteins by immunotherapy and photo-oxygenation. Immunotherapy aims to activate microglial clearance of amyloids by antibodies, either made intrinsically by active immunization of peptides or administered by passive immunization of amyloid-specific antibodies. Photo-oxygenation activates microglial clearance through oxygenation of amyloid aggregates, using photocatalysts that bind to the cross β structures common in amyloid aggregates. While immunotherapies require specific peptides or antibodies for each protein, the photocatalyst can target both Aβ and tau at the same time.

Alzheimer disease (AD) is a neurodegenerative disorder pathologically characterized by the aggregation of two amyloid proteins, amyloid-β (Aβ) and tau. These two proteins both form amyloid aggregates with cross β-sheet structures, which are toxic to neurons and are associated with AD pathogenesis. Therefore, therapeutic strategies to decrease the concentration of amyloid within the brain are considered to be effective for AD. For example, immunotherapies targeting Aβ and tau have recently gained attention, because the use of several anti-Aβ antibodies was found to enhance Aβ clearance from the brain by microglial cells (Schenk et al., 1999; Sevigny et al., 2016; Figure 1, top). Although the clinical trials of Aβ antibodies for AD patients have started, the efficient delivery of these antibodies to the brain is still an issue and the development of alternative strategies using materials with more efficient delivery may result in greater therapeutic effects.

As an alternative therapeutic strategy that is comparable to immunotherapies, we have developed the photo-oxygenation system that enhances the clearance and suppresses the aggregation of amyloid proteins (Taniguchi et al., 2014, 2016; Ni et al., 2018; Suzuki et al., 2019; Nagashima et al., 2021; Ozawa et al., 2021; Figure 1, bottom). Photo-oxygenation uses a small compound, the photocatalyst, which binds to the cross β-sheet structures of amyloid aggregates. Photocatalysts are activated by light irradiation, generating singlet oxygen when they relax to their ground state, which results in the selective photo-oxygenation of amyloid aggregates. We showed that this technology can successfully photo-oxygenate Aβ and tau aggregates, and decrease them by inhibiting Aβ and tau aggregation both *in vitro* and *in vivo*. Furthermore, more notably, photo-oxygenation enhanced the clearance of Aβ from the brain, which was confirmed by the microglia-dependent rapid clearance of photo-oxygenated Aβ aggregates. These results suggested that photo-oxygenation has great potential as a novel therapeutic strategy against AD, which is comparable to immunotherapies (Figure 1, bottom), and we introduce this technology here.

## AD and Glia

### Amyloid Pathology in AD

AD is the most common neurodegenerative disorder, and it is estimated that AD patients will continue to increase, reaching 78 million worldwide by 2030 (Alzheimer’s Disease International, 2021). The main symptoms of AD include cognitive impairment and memory loss, which greatly impact daily life. The pathological characteristics of AD are two types of protein accumulations: senile plaques consisting of Aβ, and neurofibrillary tangles including tau. Aβ is produced by the cleavage of the transmembrane region of amyloid precursor protein (APP) by β- and γ-secretases (Kikuchi et al., 2017). Tau is a microtubule-binding protein expressed in the axons of neurons. Aβ and tau are both categorized as amyloid proteins, as they form amyloid aggregates with cross β-sheet structures in pathological conditions, including AD. As the aggregation of these proteins is considered to cause neurodegeneration, Aβ and tau aggregates are possible targets for disease-modifying therapies against AD.

### Microglia in AD Pathology

Another feature in AD patients’ brains is neuroinflammation caused by microglial activation. Aggregated Aβ and tau are known to trigger an inflammatory response by microglia. Microglia that are activated by Aβ oligomers engulf synapses and mediate synaptic loss in AD (Hong et al., 2016). This is supported by the negative correlations between activated microglia and brain functions observed in AD patients (Fan et al., 2015). Furthermore, recent studies suggest that inflammation may enhance the aggregation of Aβ, *via* activated inflammasome-related proteins acting as a core for Aβ aggregation (Venegas et al., 2017). The involvement of microglia in AD pathology is also supported by research showing that several risk genes found from genome-wide association studies of AD, such as *TREM2* (triggering receptor expressed on myeloid cells-2), are mainly expressed in microglia (Gosselin et al., 2017). TREM2 is a transmembrane protein that acts as a receptor for Aβ, inducing intracellular signaling and resulting in an inflammatory response by microglia (Zhao et al., 2018). Recent studies have also demonstrated the association between TREM2 and tau pathology (Bemiller et al., 2017). Thus, inflammatory responses of microglia triggered by Aβ and tau aggregates may contribute to both neuronal dysfunction and amyloid aggregation in AD patients, aggravating AD pathology. On the other hand, microglia are also involved in the clearance of Aβ and tau, which may contribute to reducing the spread of AD pathology. Impairment of Aβ uptake and clearance by microglia increases Aβ pathology and accelerates cognitive decline (Ren et al., 2022). Microglial autophagy attenuates tau pathology through the facilitation of tau clearance (Xu et al., 2021). Whereas the ability of microglia to clear Aβ is widely known, the overaccumulation of Aβ leads to the failure of its clearance owing to microglial death as a result of inflammation. Consequently, decreased clearance of Aβ by microglia is common in AD patients. Thus, these data indicate that microglia are deeply involved in the amyloid pathogenesis of AD.

### Immunotherapy *via* Microglial Phagocytosis

As microglia contribute to AD pathogenesis, a promising therapeutic strategy is the modulation of amyloid pathology using microglia. As a method to enhance the microglial clearance of Aβ and tau for AD therapy, a recently highlighted strategy is immunotherapy using either active or passive immunization (Figure 1, top). Active immunotherapies involve the injection of fragments or the full form of Aβ or tau, to enhance the production of anti-Aβ or anti-tau antibodies. Aβ peptide vaccination showed a decrease in Aβ pathology (Schenk et al., 1999). A decrease in plaques together with the phagocytosis of Aβ by microglia was observed after the injection of Aβ peptides into AD patients, which also lead to less tau pathology in the plaque-removed areas (Nicoll et al., 2019). In passive immunotherapies, anti-Aβ or anti-tau antibodies are directly injected into patients. Studies have shown that the administration of Aβ antibodies mediates the microglial phagocytosis of Aβ aggregates through Fc receptors and that microglia degrade Aβ aggregates after phagocytosis (Bard et al., 2000; Sevigny et al., 2016). The recent United States Food and Drug Administration’s approval of aducanumab, a human monoclonal antibody that binds to aggregated Aβ, was highlighting news. Aducanumab was shown to reduce Aβ plaques in AD patients (Sevigny et al., 2016). However, there are still some controversies, such as its safety, as amyloid-associated imaging abnormalities are observed in treated patients, which may lead to associated side effects (Salloway et al., 2022). Furthermore, its high price also affects patients, families, and even insurance and taxpayers. Many drugs for AD immunotherapies are still undergoing clinical trials, and may face similar problems, as well as other concerns, such as their effective delivery through the blood-brain barrier (BBB). Therefore, there are still many challenges to overcome for the wider application of AD immunotherapies.

## Photo-Oxygenation

### Photocatalysts

**Figure 2:**
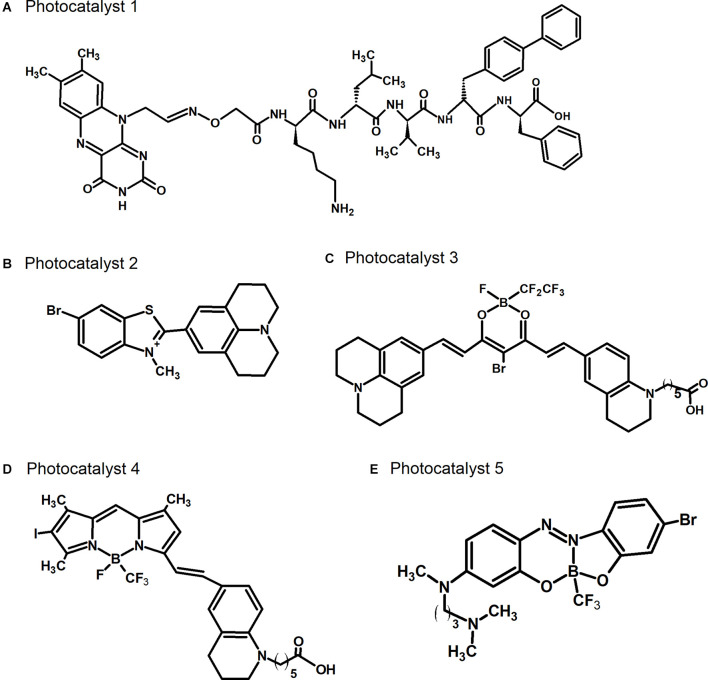
Chemical structures of photocatalysts. **(A)** Photocatalyst 1 described in Taniguchi et al. (2014). **(B)** Photocatalyst 2 described in Taniguchi et al. (2016). **(C)** Photocatalyst 3 described in Ni et al. (2018). **(D)** Photocatalyst 4 described in Suzuki et al. (2019). **(E)** Photocatalyst 5 described in Nagashima et al. (2021).

As a novel AD therapy that is comparable to immunotherapies, we have developed the photo-oxygenation technology, which can attach oxygen atoms artificially to amyloid fibrils, such as those of Aβ and tau, because oxidated Aβ showed the low aggregation potency. This strategy involves small compounds, photocatalysts (Figure 2). Photocatalyst **1**, which we developed first (Figure 2A), successfully photo-oxygenated Aβ (Taniguchi et al., 2014), but its application was limited owing to the low selectivity of the reaction. Improvements were subsequently made to increase the amyloid selectivity of the photocatalysts, based on selective fluorescent probes that bind to amyloid (Taniguchi et al., 2016). These improved photocatalysts **2**, **3**, **4**, and **5** can only be activated by light irradiation when bound to the cross β-sheet structures of Aβ or tau. The irradiated photocatalysts generate singlet oxygen when relaxing to their ground state, leading to the selective photo-oxygenation of amyloid fibrils. After further improvements, photocatalyst **3** that could be activated by near-infrared light was developed, and *in vivo* photo-oxygenation of Aβ was confirmed (Figure 2C; Ni et al., 2018). Another photocatalyst, photocatalyst **4**, which has higher photo-oxygenating activity than the previous catalysts, was also developed (Figure 2D; Suzuki et al., 2019). Photocatalyst **4** showed high photo-oxygenation activity for both Aβ and tau (Ozawa et al., 2021), indicating the high potential of photo-oxygenation as a therapeutic strategy for AD, by targeting both Aβ and tau. Photo-oxygenation using these catalysts progressed at histidine and methionine residues in Aβ and tau.

### Effects of Photo-Oxygenation

To investigate the effects of photo-oxygenation, we first analyzed the fibrillization of Aβ and tau. Photo-oxygenation clearly inhibited the fibrillization process of both proteins, and resulted in the reduction of cell toxicity caused by Aβ aggregates (Ni et al., 2018). More importantly, photo-oxygenation of aggregated seeds in the fibrillization process using photocatalyst **4** inhibited further aggregation caused by the incorporation of tau monomers into seeds (Suzuki et al., 2019). Tau pathology is known to spread through the brain in an ordered pattern, by the prion-like cell-to-cell propagation of aggregated tau seeds, and this propagation pathway is considered to be a therapeutic target of AD. Thus, this inhibitory effect of photo-oxygenation can lead to the suppression of the spread of tau pathology. Experiments using cultured cells transfected with unmodified or photo-oxygenated aggregated tau seeds showed that photo-oxygenation suppressed intracellular tau aggregation induced by seeds (Suzuki et al., 2019), indicating the successful inhibition of the cell-to-cell propagation of tau aggregation.

To further investigate the effects of Aβ and tau photo-oxygenation *in vivo*, photo-oxygenation using photocatalyst **4** was performed in a living AD model mouse, *App^NLGF/NLGF^*, which is a knock-in mouse of human Aβ harboring familial AD mutations, demonstrating the age-dependent deposition of Aβ in the brain (Saito et al., 2014). Once a day, photocatalyst **4** was directly injected into the hippocampus of this mouse, followed by light irradiation. Repeating this process for 7 days showed that Aβ aggregates were successfully photo-oxygenated even in the brains of living mice (Ozawa et al., 2021). Notably, we found that photo-oxygenation reduced Aβ levels in the brain (Ozawa et al., 2021). As one reason for this Aβ reduction, we also demonstrated that photo-oxygenated Aβ was rapidly cleared from the brain. Moreover, the rapid clearance of photo-oxygenated Aβ was canceled in mice in which microglia were depleted by Pexidartinib, a CSF-1 receptor inhibitor, inducing microglial apoptosis, suggesting that microglia are the responsible cell type for the rapid clearance of photo-oxygenated Aβ. Analysis using the mouse microglial cell line MG6 also showed enhanced degradation of photo-oxygenated Aβ, which was abolished by inhibition of the lysosomal pathway. However, no rapid degradation was observed in the human astrocytoma cell line H4, strongly supporting the idea that degrading enzymes in the lysosomes of microglia are responsible for the clearance of photo-oxygenated Aβ.

Thus, taking together the inhibitory effects of photocatalyst **4** on fibrillization, it is noteworthy that the reduction of Aβ by photo-oxygenation *in vivo* is attributed to both the inhibition of aggregation and the microglia-dependent promotion of Aβ clearance. These effects of photo-oxygenation on amyloid proteins are comparable to immunotherapy using antibodies, and hence photo-oxygenation of Aβ is a promising therapeutic strategy for AD.

### Therapeutic Potential of Photo-Oxygenation

The conventional photocatalysts **2**, **3**, and **4** are unable to cross the BBB due to their high molecular weight and ionic structures (quaternary ammonium/carboxylate), and can only be administered to the brain through direct injection. We recently developed the new photocatalyst **5**, with a substantially lower molecular weight than the conventional photocatalysts, which hence has higher BBB permeability, enabling its delivery into the brain by non-invasive administration methods, such as intravenous injection (Figure 2E; Nagashima et al., 2021). Photocatalyst **5** also oxygenates amyloid selectively, which is achieved by the inhibition of its bending mobility in the bound state with amyloids, followed by selective generation of singlet oxygen when it is irradiated with light. We showed that non-invasive light irradiation of the brain from outside of the skull of living AD model mice in which photocatalyst **5** was administered by intravenous injection led to the oxygenation of Aβ in the brain, indicating successful non-invasive photo-oxygenation *in vivo* without any surgery. In addition, we found a decrease in the amount of Aβ in the brain even by this non-invasive photo-oxygenation method, to a level comparable to photo-oxygenation by direct brain injection of photocatalyst **4** (Nagashima et al., 2021). It has been considered that photo-oxygenation leads to the inhibition of aggregation and the enhancement of microglia-dependent Aβ clearance, resulting in a decrease in Aβ levels. These results strongly suggest the potential of photo-oxygenation as an effective and non-invasive therapy, advancing towards its clinical application for AD therapy. We have also shown that Aβ derived from the brains of AD patients can also be photo-oxygenated (Ozawa et al., 2021), supporting its potential for human application.

## Conclusion

As described, we clearly showed that the selective addition of oxygen to amyloid aggregates by photo-oxygenation catalysts leads to a reduction in toxic amyloid levels in the brain, by two potential effects: the inhibition of amyloid formation, and the promotion of amyloid clearance by microglia (Figure 1, bottom). Moreover, the improvement and development of photocatalysts for the non-invasive photo-oxygenation of amyloids *in vivo* also showed promise for human application. These results indicate that photo-oxygenation is comparable to immunotherapy using antibodies against amyloids, such as Aβ and tau. Furthermore, these photocatalysts can photo-oxygenate both Aβ and tau at the same time, as was shown for photocatalyst **4** (Suzuki et al., 2019; Ozawa et al., 2021), and this is considered as an advantage over antibodies, which can react only to a single target. However, these are still some problems we should overcome. For example, it is difficult to reach light energy in deep areas of the human brain. Thus, although we still need further improvements for human application, we believe that the proof of concept of our technology as an AD therapeutic strategy is provided.

Several neurodegenerative disorders other than AD, such as Parkinson’s disease and amyotrophic lateral sclerosis, are also caused by the deposition of amyloid proteins, such as α-synuclein and TAR DNA-binding protein 43 kDa, respectively. In addition, several systemic amyloidoses cause the formation of amyloid deposits in several peripheral organs, such as the heart, kidney, and liver. These amyloid deposits in each disease comprise amyloid fibrils with cross β-sheet structures. Because our photocatalysts recognize the cross β-sheet structure and promote photo-oxygenation, this photo-oxygenation strategy may hence be widely applicable to these various proteinopathies with amyloid deposition. We intend to apply our strategies to various amyloids causing proteinopathies in the future.

In conclusion, amyloid-selective photo-oxygenation based on the inhibition of amyloid aggregation and the enhancement of amyloid clearance by microglia is expected to be an innovative therapeutic strategy for the various proteinopathies, including AD. We plan to further improve our technology, to enable its practical use in the future.

## Author Contributions

IT, HN, and YH wrote the draft and revised it. YS, MK, and TT were involved in discussing, drafting, and editing the manuscript. All authors contributed to the article and approved the submitted version.

## Conflict of Interest

The authors declare that the research was conducted in the absence of any commercial or financial relationships that could be construed as a potential conflict of interest.

## Publisher’s Note

All claims expressed in this article are solely those of the authors and do not necessarily represent those of their affiliated organizations, or those of the publisher, the editors and the reviewers. Any product that may be evaluated in this article, or claim that may be made by its manufacturer, is not guaranteed or endorsed by the publisher.
